# Supplementary material for: Social and maternal behavior in mesoderm specific transcript (Mest)-deficient mice
Source: PLoS One. 2022 Jul 22;17(7):e0271913. doi: 10.1371/journal.pone.0271913 (PMC9307168; doi:10.1371/journal.pone.0271913)
Supplement: S1 Table — (DOCX) [file pone.0271913.s001.docx]

**Supplemental Table 1. Tail Suspension Test**

|  |  |  | **STILL** | | | | MOVING | | |  |
| --- | --- | --- | --- | --- | --- | --- | --- | --- | --- | --- |
| **ID** | **genotype** | **sex** | **Latency [s]** | **Total duration [s]** | **Mean [s]** | **Total number** | **Total duration [s]** | **Mean [s]** | **Total number** | **note** |
| 7183 | n/a | n/a | n/a | n/a | n/a | n/a | n/a | n/a | n/a | **tail climber** |
| 7189 | n/a | n/a | n/a | n/a | n/a | n/a | n/a | n/a | n/a | **tail climber** |
| 7190 | n/a | n/a | n/a | n/a | n/a | n/a | n/a | n/a | n/a | **tail climber** |
| 7191 | n/a | n/a | n/a | n/a | n/a | n/a | n/a | n/a | n/a | **tail climber** |
| 7197 | n/a | n/a | n/a | n/a | n/a | n/a | n/a | n/a | n/a | **tail climber** |
| 7198 | n/a | n/a | n/a | n/a | n/a | n/a | n/a | n/a | n/a | **tail climber** |
| 7202 | n/a | n/a | n/a | n/a | n/a | n/a | n/a | n/a | n/a | **tail climber** |
| 7217 | n/a | n/a | n/a | n/a | n/a | n/a | n/a | n/a | n/a | **tail climber** |
| 7218 | n/a | n/a | n/a | n/a | n/a | n/a | n/a | n/a | n/a | **tail climber** |
| 7230 | n/a | n/a | n/a | n/a | n/a | n/a | n/a | n/a | n/a | **tail climber** |
| 7235 | n/a | n/a | n/a | n/a | n/a | n/a | n/a | n/a | n/a | **tail climber** |
| 7237 | n/a | n/a | n/a | n/a | n/a | n/a | n/a | n/a | n/a | **tail climber** |
| 7199 | n/a | n/a | n/a | n/a | n/a | n/a | n/a | n/a | n/a | **tail climber** |
| 7200 | n/a | n/a | n/a | n/a | n/a | n/a | n/a | n/a | n/a | **tail climber** |
| 7201 | n/a | n/a | n/a | n/a | n/a | n/a | n/a | n/a | n/a | **tail climber** |
| 7203 | n/a | n/a | n/a | n/a | n/a | n/a | n/a | n/a | n/a | **tail climber** |
| 7208 | n/a | n/a | n/a | n/a | n/a | n/a | n/a | n/a | n/a | **tail climber** |
| 7216 | n/a | n/a | n/a | n/a | n/a | n/a | n/a | n/a | n/a | **tail climber** |
| 7222 | n/a | n/a | n/a | n/a | n/a | n/a | n/a | n/a | n/a | **tail climber** |
| 7223 | n/a | n/a | n/a | n/a | n/a | n/a | n/a | n/a | n/a | **tail climber** |
| 7231 | n/a | n/a | n/a | n/a | n/a | n/a | n/a | n/a | n/a | **tail climber** |
| 7232 | n/a | n/a | n/a | n/a | n/a | n/a | n/a | n/a | n/a | **tail climber** |
| 7234 | n/a | n/a | n/a | n/a | n/a | n/a | n/a | n/a | n/a | **tail climber** |
| 7238 | n/a | n/a | n/a | n/a | n/a | n/a | n/a | n/a | n/a | **tail climber** |
| 7184 | n/a | n/a | n/a | n/a | n/a | n/a | n/a | n/a | n/a | **tail climber** |
| 7185 | n/a | n/a | n/a | n/a | n/a | n/a | n/a | n/a | n/a | **tail climber** |
| 7192 | n/a | n/a | n/a | n/a | n/a | n/a | n/a | n/a | n/a | **tail climber** |
| 7210 | n/a | n/a | n/a | n/a | n/a | n/a | n/a | n/a | n/a | **tail climber** |
| 7214 | n/a | n/a | n/a | n/a | n/a | n/a | n/a | n/a | n/a | **tail climber** |
| 7219 | n/a | n/a | n/a | n/a | n/a | n/a | n/a | n/a | n/a | **tail climber** |
| 7220 | n/a | n/a | n/a | n/a | n/a | n/a | n/a | n/a | n/a | **tail climber** |
| 7228 | n/a | n/a | n/a | n/a | n/a | n/a | n/a | n/a | n/a | **tail climber** |
| 7229 | n/a | n/a | n/a | n/a | n/a | n/a | n/a | n/a | n/a | **tail climber** |
| 7244 | n/a | n/a | n/a | n/a | n/a | n/a | n/a | n/a | n/a | **tail climber** |
| 7186 | n/a | n/a | n/a | n/a | n/a | n/a | n/a | n/a | n/a | **tail climber** |
| 7194 | n/a | n/a | n/a | n/a | n/a | n/a | n/a | n/a | n/a | **tail climber** |
| 7204 | n/a | n/a | n/a | n/a | n/a | n/a | n/a | n/a | n/a | **tail climber** |
| 7211 | n/a | n/a | n/a | n/a | n/a | n/a | n/a | n/a | n/a | **tail climber** |
| 7221 | n/a | n/a | n/a | n/a | n/a | n/a | n/a | n/a | n/a | **tail climber** |
| 7224 | n/a | n/a | n/a | n/a | n/a | n/a | n/a | n/a | n/a | **tail climber** |
| 7225 | n/a | n/a | n/a | n/a | n/a | n/a | n/a | n/a | n/a | **tail climber** |
| 7236 | n/a | n/a | n/a | n/a | n/a | n/a | n/a | n/a | n/a | **tail climber** |
| 7239 | n/a | n/a | n/a | n/a | n/a | n/a | n/a | n/a | n/a | **tail climber** |
| 7240 | n/a | n/a | n/a | n/a | n/a | n/a | n/a | n/a | n/a | **tail climber** |
| 7241 | n/a | n/a | n/a | n/a | n/a | n/a | n/a | n/a | n/a | **tail climber** |
| 7243 | n/a | n/a | n/a | n/a | n/a | n/a | n/a | n/a | n/a | **tail climber** |
| 7245 | n/a | n/a | n/a | n/a | n/a | n/a | n/a | n/a | n/a | **tail climber** |
| **7182** | **WT** | **F** | **47.12** | **113.04** | **5.95** | **19** | **186.96** | **9.84** | **19** | okay |
| **7193** | **WT** | **M** | **52.76** | **161.65** | **7.35** | **22** | **138.35** | **6.29** | **22** | okay |
| **7212** | **WT** | **M** | **40.23** | **153.16** | **6066** | **23** | **146.84** | **6.38** | **23** | okay |
| **7226** | **WT** | **M** | **29.83** | **162.60** | **10.84** | **15** | **137.40** | **9.16** | **15** | okay |
| **7227** | **WT** | **M** | **63.38** | **155095** | **7.43** | **21** | **144.05** | **6.55** | **22** | okay |
| **7233** | **pKO** | **F** | **60.71** | **164.08** | **9.65** | **17** | **135.92** | **8.00** | **17** | okay |
| **7213** | **pKO** | **M** | **36.96** | **177.89** | **7.12** | **25** | **122.11** | **4.88** | **25** | okay |
| **7215** | **pKO** | **M** | **41.50** | **134.24** | **8.95** | **15** | **165.76** | **11.05** | **15** | okay |
|  |  |  |  |  |  |  |  |  |  |  |
|  | **Mean WT** | | **46.67** | **149.28** | **7.64** | **20.00** | **150.72** | **7.64** | **20.20** |  |
|  | **Mean pKO** | | **46.39** | **158.74** | **8.57** | **19.00** | **141.26** | **7.98** | **19.00** |  |
|  | **TTEST; pvalue** | | **0.98** | **0.56** | **0.49** | **0.74** | **0.56** | **0.85** | **0.70** |  |
