# Supplementary material for: Social and maternal behavior in mesoderm specific transcript (Mest)-deficient mice
Source: PLoS One. 2022 Jul 22;17(7):e0271913. doi: 10.1371/journal.pone.0271913 (PMC9307168; doi:10.1371/journal.pone.0271913)
Supplement: S1 Raw images — (PDF) [file pone.0271913.s003.pdf]

S1 Raw Images_Anunciado-Koza et al

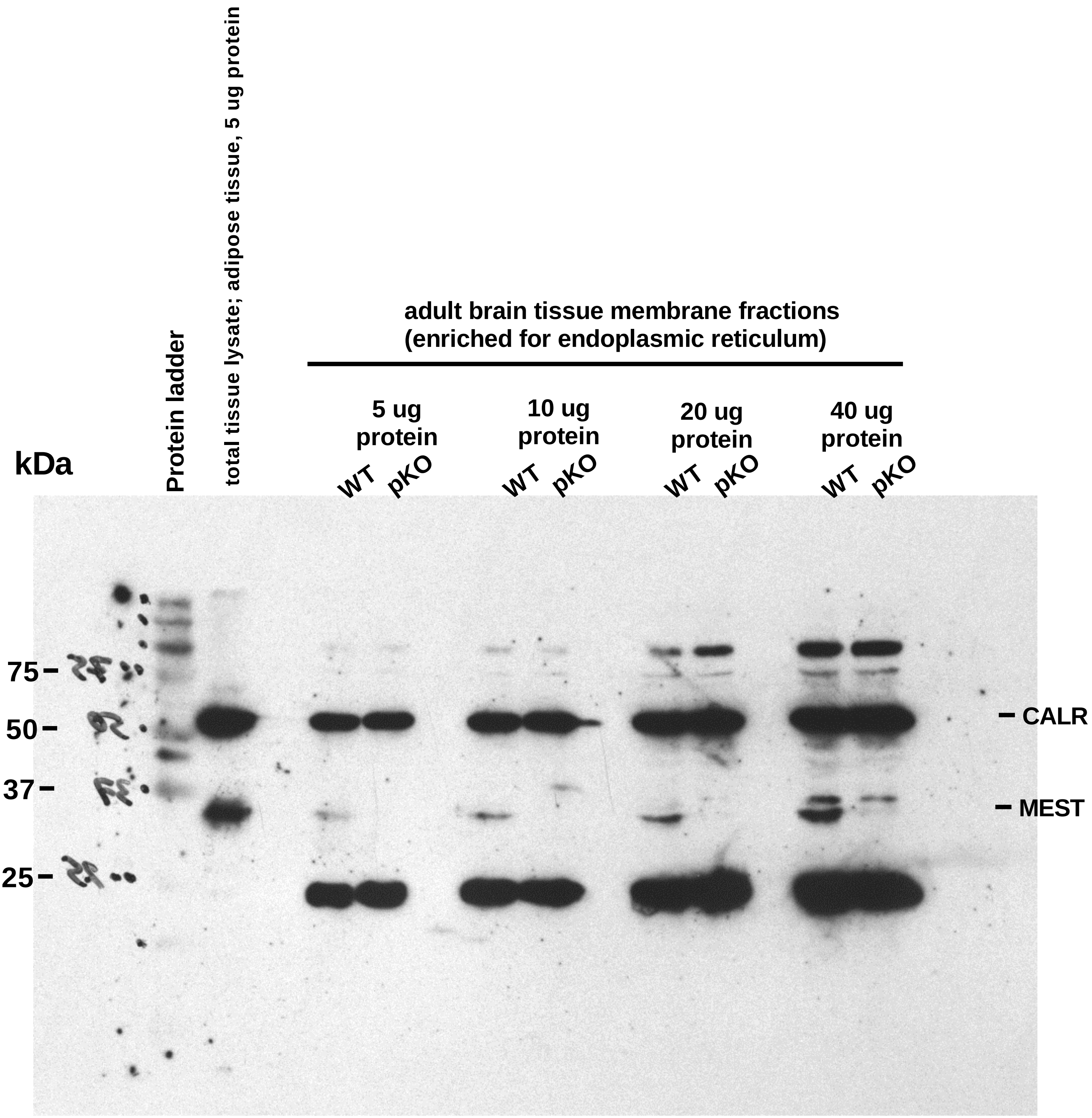

12% PAGE-transfer to nitrocellulose membrane
developed via chemilumenescence on film
film scanned for digital image using UVP GelDoc-It Imager
